# Prediction of activity in eyes with macular neovascularization due to age-related macular degeneration using deep learning

**DOI:** 10.1038/s41433-023-02805-4

**Published:** 2023-10-26

**Authors:** Giulia Corradetti, Nadav Rakocz, Jeffrey N. Chiang, Oren Avram, Ahmed Roshdy Alagorie, Muneeswar Gupta Nittala, Ayesha Karamat, David S. Boyer, David Sarraf, Eran Halperin, SriniVas Sadda

**Affiliations:** 1 Doheny Eye Institute, Pasadena, CA USA; 2 Department of Ophthalmology, David Geffen School of Medicine, University of California-Los Angeles, Los Angeles, CA USA; 3 Department of Computer Science, University of California-Los Angeles, Los Angeles, CA USA; 4 Department of Computational Medicine, University of California-Los Angeles, Los Angeles, CA USA; 5 Department of Anesthesiology and Perioperative Medicine, University of California-Los Angeles, Los Angeles, CA USA; 6 Department of Ophthalmology, Faculty of Medicine, Tanta University, Tanta, Egypt; 7 Retina-Vitreous Associates Medical Group, Beverly Hills, CA USA; 8 Retinal Disorders and Ophthalmic Genetics Division, University of California-Los Angeles, Los Angeles, CA USA; 9 Department of Human Genetics, University of California-Los Angeles, Los Angeles, CA USA

**Keywords:** Prognostic markers, Risk factors, Education

Optical coherence tomography (OCT) technology has been particularly transformative for the clinical management of age-related macular degeneration (AMD) as it provides unprecedented depth resolution and 3-D visualization of the retina, allowing for the detection of fluid at different levels within the retina.

Identification of intraretinal fluid (IRF), subretinal fluid (SRF), and sub-retinal pigment epithelium (sub-RPE) fluid is commonly used to determine the presence of disease activity, which is used to guide the frequency of anti-angiogenic therapy. Structural OCT can also capture the location of the macular neovascularization (MNV) which can be sub-RPE (Type 1 MNV), subretinal (Type 2), intraretinal (Type 3), or mixed Type 1/2 [1]. However, the detailed morphology of the neovascular network cannot be resolved on structural OCT. With the advent of OCT-Angiography (OCTA), microvascular morphologic details of the MNV lesion and the surrounding choriocapillaris can be captured. A number of small studies have evaluated the OCTA features of MNV lesions at various stages in their evolution and in response to therapy [2–4].

Other researchers have gone on to speculate regarding which OCTA features could potentially be used to guide retreatment decisions for neovascular AMD [1–7]. While one might question the clinical value of identifying markers of activity on OCTA given that activity can be assessed on the concomitant structural OCT available with every OCTA acquisition, identification of OCTA features of MNV activity may provide new insights into the pathophysiology and maturation of these lesions. In addition, OCTA features of MNV activity could potentially provide predictive biomarkers of long-term visual and anatomical outcomes and could predict future activity in eyes with nonexudative MNV without fluid on structural OCT.

Machine learning approaches have been used to perform classification of retinal diseases on various retinal imaging modalities, including OCT [8].

We have evaluated several machine learning algorithms for detecting neovascular disease activity on en face OCTA images only by training the model using activity as determined by the presence of exudation on structural OCT volumes. Our objective was to assess whether we could use en face OCTA images alone to detect disease activity in MNV eyes.

In this retrospective analysis we included 637 en face OCTA scans from 97 patients, who were diagnosed with neovascular AMD (MNV type 1 or 2) and imaged using a 6 × 6 mm pattern centered on the fovea using the RTvue-XR Avanti SD-OCTA (Optovue, Inc, Fremont, CA) device at the Stein Eye Institute UCLA and Retina-Vitreous Associates Medical Group, both in Los Angeles, CA, United States.

**Fig. 1:**
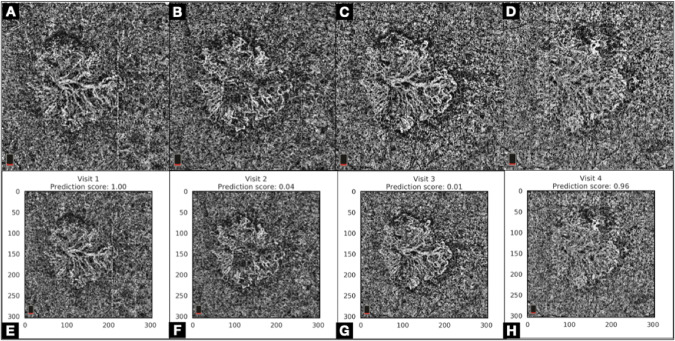
Choriocapillaris en face OCTA and prediction scores to detect activity of macular neovascularization. Upper Row (**A**–**D**). Choriocapillaris en face OCTA in a case of Type 1 Macular Neovascularization (MNV) at four consecutive monthly visits. The choriocapillaris en face OCTA images (**A**, **B**, **C**, **D**) show a sea-fan appearance of the MNV, which activity is difficult to assess based on the en face OCTA images only. Lower Row (**E**–**H**). Prediction Score generated for each choriocapillaris en face OCTA using machine learning algorithms, showing an overall poor predictive value to detect activity of MNV based on the en face OCTA images alone.

The visualization of the MNV lesions on en face OCTA was obtained using a customized 10-micron thick slab (Fig. 1), for which the segmentation boundaries were manually adjusted to display the maximum extent of the MNV lesion [2, 9].

Multiple machine learning models were trained to classify the presence of MNV activity by OCTA imaging, using the presence of fluid on the structural OCT as the ground truth evidence for activity.

The algorithms that were tested included: 1) a pretrained Resnet18 deep learning architecture (referred to as ResnetPre), which was previously shown to achieve good results with the interpretation of retinal medical images [10], even in the setting of a small sample size; 2) a randomly initialized Resnet18 trained from scratch (referred to as Resnet-Scratch); 3) a logistic regression combined with dimensionality reduction in the form of principal component analysis [11–13] (referred to as LR + PCA); and 4) a simpler deep learning architecture which contains two CNN layers and three fully connected layers (referred to as SimpleCNN).

The performance of the various models was evaluated by using cross-validation and the ROC and its area under the curve. A power analysis was used to assess the effect of sample size on models’ performance by sampling an increasing number of patients from the data and repeating the analysis.

**Fig. 2:**
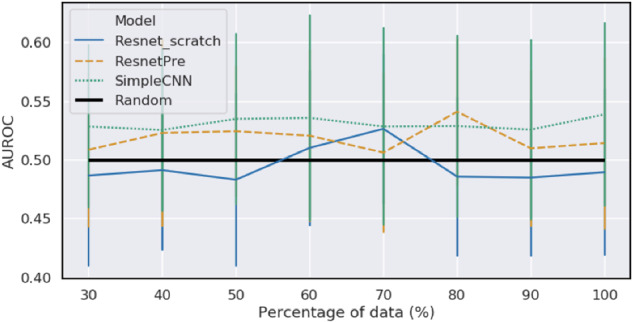
Performance (AUROC) of various machine learning models along with 95% confidence intervals when trained on portion of the data ranging from 30 to 100%. No model is ever significantly better than random. There is also no visible trend of improvement as sample size increases.

From the results of the tested models (Fig. 2) it was apparent that none of the models was able to produce performance that was significantly better than a random decision with the top performing model, the SimpleCNN, achieving an AUROC of 0.54 [0.39, 0.69]; a random algorithm is expected to result in an AUROC of 0.5. This would suggest that the information that is incorporated in the en face OCTA images may not be sufficient to detect the activity of MNV with high accuracy, at least with this study sample size.

The power analysis that was used also suggests that an increase in sample size is unlikely to lead to substantial improvement in performance as no positive trend is apparent in any of the models as samples increase.

A number of investigators have qualitatively inspected OCTA images from eyes with and without disease activity in an attempt to identify OCTA features predictive of such activity [3, 7, 14–17].

While all these studies demonstrated a correlation between specific OCTA features and active MNV, they were small retrospective series, and the findings have proven difficult to replicate, and more significantly when presented to a large group of experts, the relevant features could not be identified reliably [18].

Limitations of our study include its retrospective nature which may have resulted in ascertainment bias in the collection of cases. Furthermore, we focused only on the en face OCTA image because this was the OCTA data used by previous clinical studies for identifying potential predictive features. However, it is possible that volumetric OCTA data could contain additional predictive information not evident in the en face images.

The size of our training set may have impaired the performance of the algorithm [19].

However, we would contend that the algorithms we used provided results that are substantially better than random in other problems with similar sample size [10]. Moreover, we have used power analysis to show that even as sample size increases, these algorithms demonstrate no improvement in performance, and thus, even if the sample size was substantially larger we would not expect a considerable improvement in performance.

In conclusion, our analysis would suggest that the assessment of en face OCTA images alone cannot provide a reliable determination of activity of an MNV lesion at a particular point in time. Further studies using swept source OCTA and denser scan patterns are necessary to assess whether the OCTA can provide other information of prognostic value in neovascular AMD.
